# *N*-hexanoyl-sphingomyelin potentiates *in vitro* doxorubicin cytotoxicity by enhancing its cellular influx

**DOI:** 10.1038/sj.bjc.6601581

**Published:** 2004-02-17

**Authors:** R J Veldman, S Zerp, W J van Blitterswijk, M Verheij

**Affiliations:** 1 Division of Cellular Biochemistry, The Netherlands Cancer Institute, Antoni van Leeuwenhoek Hospital, Plesmanlaan 121, NL-1066 CX Amsterdam, The Netherlands; 2 Department of Radiotherapy, The Netherlands Cancer Institute, Antoni van Leeuwenhoek Hospital, Plesmanlaan 121, NL-1066 CX Amsterdam, The Netherlands

**Keywords:** chemotherapy, anthracycline, sphingolipid, potentiation, drug delivery

## Abstract

Anticancer drugs generally have intracellular targets, implicating transport over the plasma membrane. For amphiphilic agents, such as the anthracycline doxorubicin, this occurs by passive diffusion. We investigated whether exogenous membrane-permeable lipid analogues improve this drug influx. Combinations of drugs and lipid analogues were coadministered to cultured endothelial cells and various tumour cell lines, and subsequent drug accumulation in cells was quantified. We identified *N*-hexanoyl-sphingomyelin (SM) as a potent enhancer of drug uptake. Low micromolar amounts of this short-chain sphingolipid, being not toxic itself, enhanced the uptake of doxorubicin up to 300% and decreased its EC_50_ toxicity values seven- to 14-fold. *N*-hexanoyl SM acts at the level of the plasma membrane, but was found not incorporated in (isolated) lipid rafts, and artificial disruption or elimination of raft constituents did not affect its drug uptake-enhancing effect. Further, any mechanistic role of the endocytic machinery, membrane leakage or ABC-transporter-mediated efflux could be excluded. Finally, a correlation was established between the degree of drug lipophilicity, as defined by partitioning in a two-phase octanol–water system, and the susceptibility of the drug towards the uptake-enhancing effect of the sphingolipid. A clear optimum was found for amphiphilic drugs, such as doxorubicin, epirubicin and topotecan, indicating that *N*-hexanoyl-SM might act by modulating the average degree of plasma membrane lipophilicity, in turn facilitating transbilayer drug diffusion. The concept of short-chain sphingolipids as amphiphilic drug potentiators provides novel opportunities for improving drug delivery technologies.

Insufficient delivery of chemotherapeutic agents to their intended molecular targets remains a major obstacle in clinical oncology. Most anticancer drugs act intracellularly, which involves a transport step over the plasma membrane. The maximally reached intracellular drug concentration largely determines the efficacy of these compounds (Speth *et al*, 1988). Optimisation of influx, across the plasma membrane barrier of target cells, would therefore increase the therapeutic efficacy of a drug. Hydrophilic compounds are presumed to traverse the plasma membrane by relatively slow processes, such as carrier-mediated transport or endocytic uptake. More efficient in this respect are lipophilic drugs, which readily cross the lipid bilayer by passive diffusion, along their concentration gradient (Robert and Gianni, 1993; Washington *et al*, 2001). However, given the fact that anticancer drugs are generally applied systemically, maintenance of plasma solubility is an additional specification for these compounds. Vacillating between these opposite requirements, antineoplastic agents often exhibit an amphiphilic nature. Optimisation of cellular drug uptake is traditionally sought for by modification of lipophilicity. However, this often compromises interactions with its molecular target (Weiss, 1992).

The amphiphilic anthracycline doxorubicin is probably the best-studied example in drug–membrane interactions. Adsorption, insertion and flip-flop can be distinguished as consecutive stages in the transport of this compound across membranes (Regev and Eytan, 1997; Lecompte *et al*, 2002). Studies performed with model systems, such as large unilamellar vesicles, *E. coli* mutants and erythrocytes, revealed that membrane potential, pH and electrostatic forces play important roles in this (Terasaki *et al*, 1984; Nishiyama *et al*, 1992; de Wolf *et al*, 1993; Speelmans *et al*, 1997). Furthermore, hydrophobic interactions, membrane fluidity and drug lipophilicity are decisive for efficient drug import (Jedrzejczak *et al*, 1999; Schuldes *et al*, 2001). In line with these observations, it is of interest that the lipid composition of model membranes determines the efficiency of anthracycline translocation, and it is well conceivable that this also holds for natural membranes (Speelmans *et al*, 1994; Lecompte *et al*, 2002).

Plasma membrane lipid bilayers are composed of dozens of different lipid species, whose distribution is far from homogeneous due to the presence of microdomains such as lipid rafts and caveolae (Sprong *et al*, 2001; Simons and Ehehalt, 2002). The main principle underlying microdomain formation is the auto-organising capacity of sphingolipids, such as sphingomyelin (SM) and glycosphingolipids, both with themselves and in association with cholesterol. Owing to their relative insolubility in nonionic detergents, such as Triton X-100, these structures have been biochemically (operationally) defined as the detergent-resistant membrane fraction (*DRM*). The number of different types of (micro)domains are unknown, but presumably the outer leaflet of the plasma membrane should be considered as a mosaic-like patchwork with a differentiated lipid distribution (Gruenberg, 2001). This model implies local differences in lipophilicity and membrane fluidity, which in turn might determine the site and efficiency of cellular drug entry.

Several tools are available for manipulation of the plasma membrane lipid composition and distribution. Inhibitors of endogenous lipid-metabolising enzymes and cholesterol-sequestering agents are generally used (Radin *et al*, 1993; Ohvo and Slotte, 1996). Furthermore, (bacterial) lipid-metabolising enzymes can be administered to cultured cells (Veldman *et al*, 1998). In addition to these indirect approaches, lipids might also be added directly to cells. Natural double-chain lipids, however, due to their bulky hydrophobic nature, are poorly soluble and do not insert spontaneously into cellular membranes. These natural (phospho)lipids, containing fatty acids composed of 16–24 carbon atoms, are useful in liposomal drug delivery systems, but not as single molecules. The use of truncated analogues, on the other hand, improves both solubility and membrane permeability of the lipids. Typically, such analogues contain one or two truncated chains, consisting of only 2 (acetyl; C_2_), 6 (hexanoyl; C_6_) or 8 (octanoyl; C_8_) carbon atoms, and easily insert as monomers in a lipid bilayer. Being structurally closely related to their natural counterparts, these semisynthetic analogues appear to be transported and metabolised normally (Watanabe *et al*, 1999). Some lipid analogues elicit strong cellular responses. C_6_-ceramide, for example, rapidly induces apoptosis in a number of cell types (Veldman *et al*, 1998). Others, such as C_6_-SM, appear to be rather inert in this respect.

With the present *in vitro* work, we investigated whether plasma membrane lipid manipulation is a feasible strategy for increasing the cellular entry of doxorubicin and other amphiphilic drugs. Of all tested methods, treatment of cells with C_6_-SM exhibited the most prominent effect on cellular doxorubicin uptake and cytotoxicity. Since the lipid itself was nontoxic at its effective concentrations (low micromolar), C_6_-SM should be considered as a novel potentiator of doxorubicin cytotoxicity. While natural long-chain SM is a common constituent of liposomal drug formulations, in which it serves a mere structural role (Betz *et al*, 2001), the short-chain SM analogue acts directly in the drug uptake process itself. Herewith, we provide the first example of a synthetic lipid that acts as a delivery enhancer of a pharmacon. As such, this finding holds promise for *in vivo* applications.

## MATERIALS AND METHODS

### Materials

Bicinchoninic acid (BCA) protein kit, 3-[4,5-dimethylthiazol-2-yl]-2,5-diphenyl tetrazolium bromide (MTT), C_6_-ceramide (C_6_-Cer), C_8_-ceramide-1-phosphate (C_8_-Cer-1P), sphingosylphosphorylcholine, dihexanoyl-phosphatidylcholine, methyl-*β*-cyclodextrin (CDX), *S. aureus* sphingomyelinase (bSMase), polyoxyethylene 20 oleyl ether (Brij 98), sulphorhodamine 101, propidium iodide, *o*-phtaldialdehyde, bisbenzimidine, camptothecin and daunorubicin were purchased from Sigma (St Louis, MO, USA). *N*-acetyl-sphingomyelin (C_2_-SM), *N*-hexanoyl-sphingomyelin (C_6_-SM) and DL-threo-1-phenyl-2-decanoylamino-3-morpholino-1-propanol (PPMP) were from Matreya (State College, PA, USA). AlexaFluor 488 (AF488) conjugates of hydrazide, cholera toxin *β* subunit (CTB) and transferrin were from Molecular Probes (Leiden, The Netherlands). Vectashield mounting medium was from Vector Laboratories (Burlingame CA, USA) and the CytoTox 96 lactate dehydrogenase (LDH) activity assay kit was from Promega (Madison WI, USA). LK6D silica TLC plates were from Whatman (Maidstone, UK). Doxorubicin was obtained from Pharmachemie (Haarlem, The Netherlands), epirubicin from Pharmacia & Upjohn (Woerden, The Netherlands), idarubicin from Farmitalia (Milan, Italy) and topotecan from SmithKline Beecham (Conshohocken PA, USA). 1-Octanol was from Riedel-de Haën (Seelze, Germany).

### Cell culture

The following cell types were cultured in Dulbecco's modified Eagle's medium (DMEM) containing 10% (v v^−1^) foetal calf serum: primary bovine aortic endothelial cells (BAEC; passage 14–19), kindly provided by Dr A Haimovitz-Friedman (Memorial Sloan-Kettering Cancer Center, New York City NY, USA); MCF-7, KB and A431 carcinoma cells, purchased from the American Type Culture Collection (Manassas VA, USA); SV40 large-T antigen-immortalised kidney fibroblasts from *Mrp1/Mdr1a/Mdr1b* triple knockout mice and control renal fibroblasts (Wijnholds *et al*, 2000; kindly provided by Dr AH Schinkel, The Netherlands Cancer Institute, Amsterdam). Cells were subcultured weekly by trypsinisation and maintained in a water-saturated atmosphere of 10% (BAEC) or 5% (MCF-7, KB, A431 and fibroblasts) CO_2_ at 37°C. All culture media were supplemented with 100 U ml^−1^ penicillin, 100 *μ*g ml^−1^ streptomycin and 4 mM L-glutamine.

### Microscopy

For microscopic studies, BAEC were cultured to confluence on 0.5%. (w v^−1^) gelatin-coated glass coverslips. Under serum-free conditions, cells were then exposed to 50 *μ*M doxorubicin for the indicated periods of time. After washing, cells were fixed in 4% (w v^−1^) paraformaldehyde in PBS for 10 min and mounted in Vectashield on glass slides. Cells were then examined with a Zeiss Axiovert S100 inverted fluorescence microscope, employing a mercury lamp in combination with a filter set consisting of a 450–490 nm band pass excitation filter, a 510 nm beam splitter and a 520 nm long-pass emission filter. All specimens were photographed through a × 20 objective by a Zeiss AxioCam CCD camera using an exposure time of 200 ms.

### Cellular drug accumulation

For intracellular doxorubicin measurements, cells were cultured in flat-bottom 96-well plates. At confluence, cells were changed to a serum-free medium and exposed to 50 *μ*M doxorubicin during 60 min (unless indicated otherwise). After washing with ice-cold PBS, cells were lysed in 1% (w v^−1^) Triton X-100 in water. Native fluorescence intensities were then measured by a Perkin-Elmer Victor Wallac II fluorescence microplate reader, using *λ*_ex_ 485 nm and *λ*_em_ 535 nm filters. Additional probes and drugs that were tested for cellular uptake during 60 min incubations, either in the absence or presence of lipid, were hydrazide-AF488 (35 *μ*M, *λ*_ex_ 485 nm, *λ*_em_ 535 nm), sulphorodamine-101 (400 *μ*M, *λ*_ex_ 355 nm, *λ*_em_ 620 nm), propidium iodide (300 *μ*M, *λ*_ex_ 355 nm, *λ*_em_ 535 nm), *o*-phtaldialdehyde (1 mM, *λ*_ex_ 485 nm, *λ*_em_ 535 nm), topotecan (30 *μ*M, *λ*_ex_ 355 nm, *λ*_em_ 535 nm), epirubicin (50 *μ*M, *λ*_ex_ 485 nm, *λ*_em_ 535 nm), bisbenzimidine (10 *μ*M, *λ*_ex_ 355 nm, *λ*_em_ 460 nm), camptothecin (30 *μ*M, *λ*_ex_ 355 nm, *λ*_em_ 460 nm), daunorubicin (25 *μ*M, *λ*_ex_ 485 nm, *λ*_em_ 535 nm) and idarubicin (20 *μ*M, *λ*_ex_ 485 nm, *λ*_em_ 535 nm). All values were corrected for background fluorescence (or radioactivity) and for differences in protein content, as determined with the BCA assay (Smith *et al*, 1985).

### Cell viability

For viability assessment, cells were cultured in flat-bottom 96-well plates. After experimental treatments, 100 μg of the mitochondrial dehydrogenase substrate MTT was added to each well (Carmichael *et al*, 1987). Cells were then incubated for 60 min at 37°C. After centrifugation (3000 rpm, 10 min) supernatants were removed. The precipitated blue formazan products were then dissolved in 100 *μ*l DMSO and absorbencies were read in a Bio-Tek Instruments EL 340 photospectrometric microplate reader at 540 nm. Background absorbencies were subtracted and values from untreated control cells were set at 100% viability.

### Plasma membrane integrity assessment

Confluent and serum-free BAEC were incubated for 120 min in the presence of C_6_-SM and 175 *μ*M of the membrane impermeant fluid phase marker hydrazide-AF488. After washing, cells were lysed in PBS containing 1% (w v^−1^) Triton X-100 and fluorescence was measured at *λ*_ex_ 485 nm and *λ*_em_ 535 nm. Alternatively, the release of the cytosolic enzyme LDH from confluent BAEC into phenol red-free DMEM was measured. For this, plates were centrifuged (5 min, 2000 rpm) and 50 *μ*l aliquots of the supernatants were assessed for LDH activity by using the CytoTox 96 kit, according to the manufacturer's instructions. Values obtained from cells lysed in 0.8% Triton X-100 served as positive control. All data were corrected for protein contents, as determined with the BCA assay (Smith *et al*, 1985).

### Endocytosis assays

To monitor clathrin- and raft-mediated endocytic activity, confluent BAEC were allowed to take up AF488-transferrin (150 *μ*g ml^−1^) or AF488-CTB (75 *μ*g ml^−1^), respectively. Incubations were performed under serum-free conditions in the presence of 10 *μ*M C_6_-SM during 120 min. Cells were then washed and lysed in PBS containing 1% (v v^−1^) Triton X-100. Fluorescence was then measured at *λ*_ex_ 485 nm and *λ*_em_ 535 nm. Values were corrected for protein content, as determined with the BCA assay (Smith *et al*, 1985).

### Isolation of detergent-resistant membrane fraction

For each isolation, four confluent ∅ 15 cm dishes with confluent BAEC were treated as indicated. Cells were then washed twice with ice-cold PBS and harvested by scraping and centrifugation (3000 rpm, 5 min, 4°C). Pellets were resuspended in 1 ml of ice-cold 0.5% (w v^−1^) Triton X-100 in MES-buffered saline (MBS; 25 mM 2-[*N*-morpholino]ethanesulphonic acid in 150 mM NaCl, pH 6.5) (Lisanti *et al*, 1995). After 30 min on ice, 1.2 ml of MBS was added and 2 ml of this suspension was mixed with 2 ml 80% sucrose (w v^−1^) in MBS and transferred to Beckman Ultraclear 14 × 89 mm tubes. This layer was overlaid with cushions of 5 ml 30% (w v^−1^) and 3 ml 5% (w v^−1^) sucrose in MBS, respectively. Remnants of the initial suspension were used for the determination of total protein, drug and lipid contents (designated as *total*). Gradients were subjected to ultracentrifugation in an SW41 swing-out rotor (39 000 rpm, 16 h, 4°C). After each run, opaque bands of insoluble material were visible in the upper half of each gradient. Tubes were aspirated in 1 ml fractions, of which the upper six (designated as *DRM*) and the lower six were pooled (designated as *non-DRM*). Alternatively, the whole procedure was carried out at room temperature using 1% (w v^−1^) Brij 98 as detergent (Pike *et al*, 2002).

### Sphingomyelin quantification

Total lipids were extracted from the sucrose gradient fractions and dried under a stream of N_2_ (Bligh and Dyer, 1959). Glycerol-containing phospholipids were hydrolysed during an overnight incubation at 37°C in 0.2 M NaOH in methanol/chloroform (1 : 1, by vol.). The remaining lipids were then re-extracted, dried and applied onto TLC plates, which were developed at room temperature using chloroform/methanol/water (60 : 30 : 8, by vol.) as the mobile phase. Lipids were visualised by iodine vapour staining and natural SM and C_6_-SM containing spots were identified with the aid of external standards, run on the same plate. Confirmation of this identification took place by bSMase pretreatment (0.1 U ml^−1^, 2 h, 20°C) of C_6_-SM-loaded cells before the extraction. Both SM and C_6_-SM spots were scraped from the plate and subjected to a colorimetric phosphate determination, using inorganic phosphate as standard (Böttcher *et al*, 1961).

### Determination of lipophilicity

Partitioning of 10–50 *μ*M drug or probe was monitored in a two-phase system consisting of 1 ml 1-octanol and 1 ml water. After addition of the compound, tubes were vortexed and centrifuged (3000 rpm, 5 min, 20°C). Aliquots of both the organic and the aqueous phases were taken for fluorimetric quantification, as described under *Cellular drug accumulation*. As a measure of lipophilicity, the 1-octanol/water partitioning ratio was calculated for each compound and expressed on a log scale (Washington *et al*, 2001).

## RESULTS

### Cellular doxorubicin uptake and toxicity is specifically enhanced by C_6_-SM

**Figure 1:**
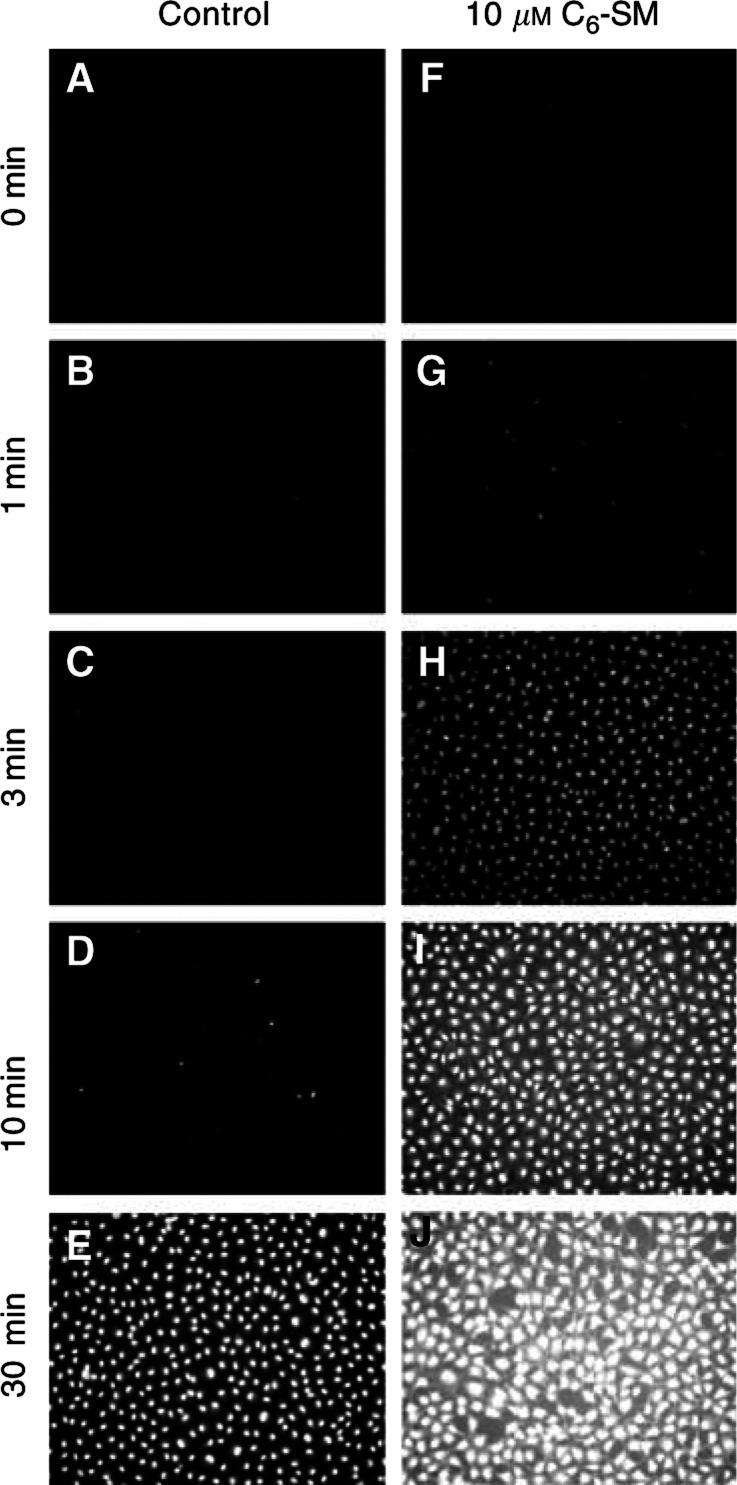
C_6_-SM enhances the cellular uptake and nuclear accumulation of doxorubicin. Confluent BAEC were incubated for the indicated periods with 50 *μ*M of doxorubicin, either in the absence (**A–E**) or presence (**F**-**J**) of 10 *μ*M C_6_-SM. Cells were then washed and fixated in 4% (v v^−1^) paraformaldehyde. After mounting, cells were examined by fluorescence microscopy, employing a filter set consisting of a 450–490 nm band pass excitation filter, a 510 nm beam splitter and a 520 nm long pass emission filter. All specimens were photographed through a × 20 objective using a 200 ms exposure time.

To study the cellular uptake of doxorubicin *in vitro*, BAEC were exposed to 50 *μ*M of the drug, and intracellular accumulation was then monitored, either by fluorescence microscopy or by fluorimetry of cell lysates. Under control conditions, intracellular doxorubicin accumulation proceeded in a time-dependent manner, starting within minutes upon addition (Figure 1a–e

and Figure 2

**Figure 2:**
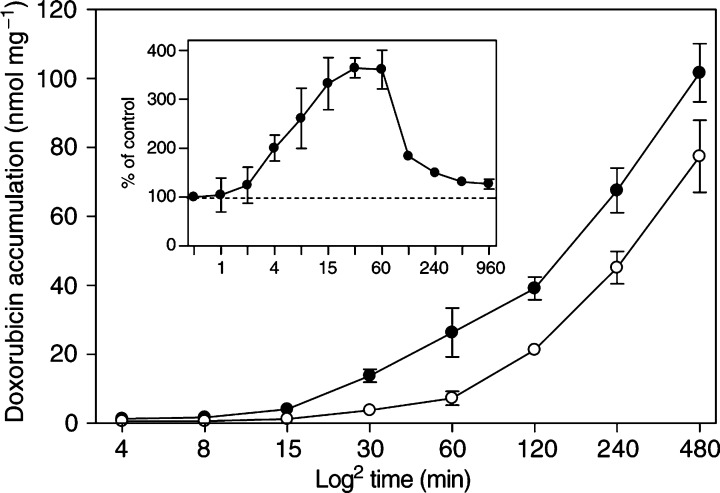
Uptake-enhancing effect of C_6_-SM on doxorubicin is most evident within the first hour. Confluent BAEC were incubated for the indicated periods with 50 *μ*M of doxorubicin, either in the absence (○) or presence (•) of 10 *μ*M C_6_-SM. Cells were then washed and lysed by 1% (w v^−1^) Triton X-100 in PBS. Employing its native fluorescent properties, doxorubicin was quantified fluorimetrically by comparison to standard amounts of the drug in the same lysis buffer. Data are expressed as nmol per mg of cellular protein (mean±s.d., *n*=6) and plotted against time (min) on a ^2^log scale. The inset shows the relative effect of C_6_-SM on doxorubicin uptake, as compared to the control cells (%±s.d.).

). This kinetic altered significantly when cells were cotreated with 10 *μ*M of C_6_-SM (Figure 1f–j and Figure 2). Under these conditions, intracellular doxorubicin was observed within the first minute and increased up to 300% over control levels for incubation periods of 15–60 min (Figure 2, inset). This relative increase gradually diminished when doxorubicin uptake was monitored for longer periods.

**Figure 3:**
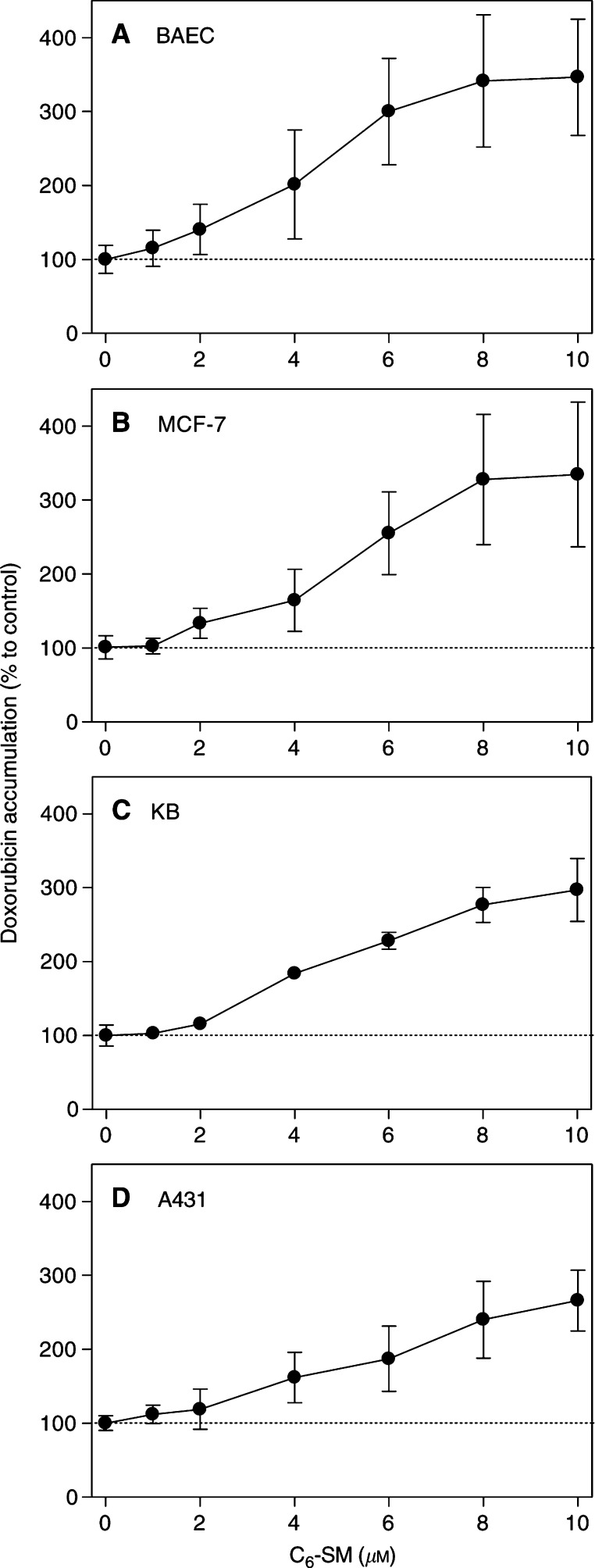
Concentration-dependency of the C_6_-SM effect on doxorubicin accumulation. BAEC (**A**), MCF-7 (**B**), KB (**C**) and A431 (**D**) cells were incubated with the indicated concentrations of C_6_-SM and 50 *μ*M doxorubicin for 60 min. After washing, cellular drug accumulation was determined fluorimetrically and all data were corrected for protein contents (*n*=6). Data are expressed as percentage (±s.d.) to the cellular doxorubicin accumulation under control conditions (50 *μ*M drug, no lipid).

The observed doxorubicin uptake-enhancing effect of C_6_-SM in BAEC was clearly concentration-dependent (Figure 3a

). A significant increase in drug uptake was seen at lipid concentrations as low as 2 *μ*M. However, a maximum increase of 300% over control values was reached at 6 *μ*M of C_6_-SM. No further increases were observed at higher concentrations. Comparable results were obtained in a panel of human doxorubicin-sensitive tumour cell lines: MCF-7 (mammary adenocarcinoma), KB (oesophagal carcinoma) and A431 (epidermoid carcinoma) cells (Figures 3b–d).

**Figure 4:**
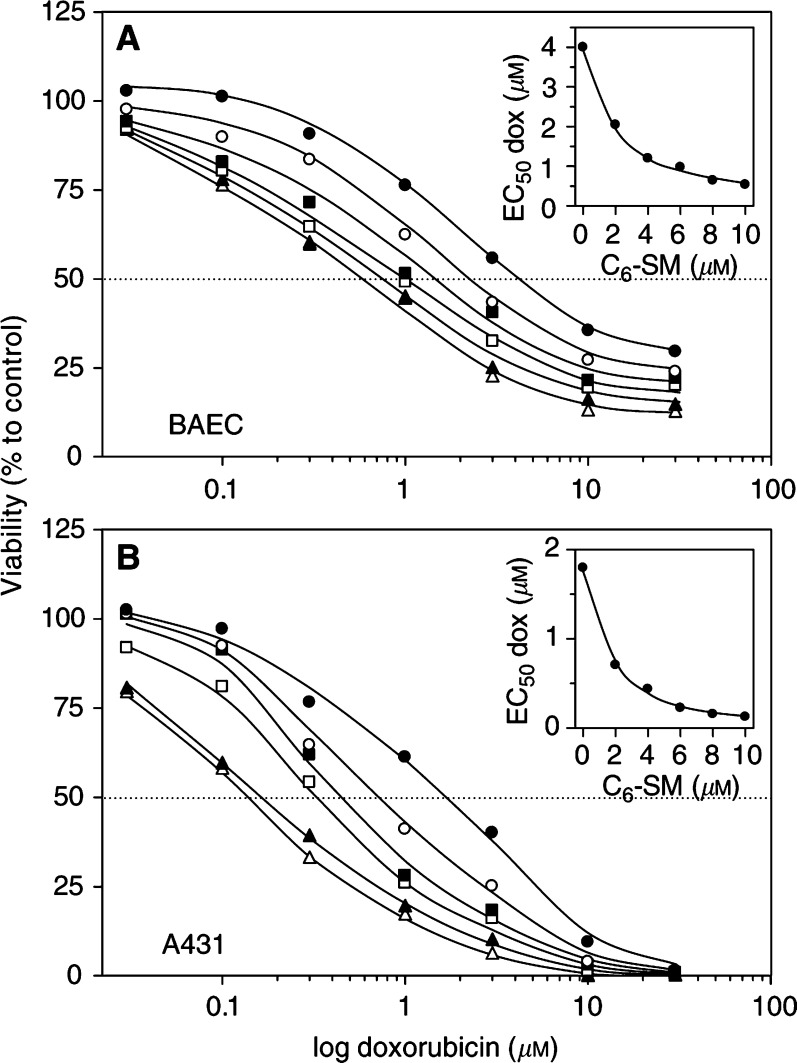
C_6_-SM potentiates the cytotoxic effect of doxorubicin at low micromolar concentrations. BAEC (**A**) and A431 (**B**) cells were incubated with various concentrations of doxorubicin, combined with 0 *μ*M (•), 2 *μ*M (○), 4 *μ*M (▪), 6 *μ*M (□), 8 *μ*M (▴) or 10 *μ*M (▵) C_6_-SM. After 60 min, cells were washed and kept in culture for another 48 h. Cell viability was then assessed by the MTT assay. Data are expressed as mean percentages (*n*=3) to untreated control cells (no drug, no lipid; set at 100% viability). The insets show the doxorubicin EC_50_ values of the viability curves plotted against the administered C_6_-SM concentrations.

To test whether increased intracellular concentrations of doxorubicin correlated with increased cytotoxicity, we allowed BAEC and tumour cell lines to accumulate doxorubicin for 60 min in the presence of varying concentrations of C_6_-SM. After washing away remaining extracellular drug and lipid, cells were cultured for another 48 h. Viability was then assessed by the MTT assay, which relies on the fact that only mitochondria of viable cells have the capacity to convert the MTT substrate (Carmichael *et al*, 1987). A concentration series of doxorubicin resulted in typical sigmoid survival curves for BAEC and A431 cells (Figures 4a and b

). Curves of cells that accumulated doxorubicin in the presence of C_6_-SM shifted leftward, indicating increased cell death. The doxorubicin EC_50_ value for BAEC toxicity shifted from 4 *μ*M in the absence of C_6_-SM to 0.6 *μ*M for cells treated with 10 *μ*M of C_6_-SM, indicating a seven-fold increase in sensitivity. In A431 cells, C_6_-SM shifted back doxorubicin EC_50_ values from 1.8 to 0.13 *μ*M, thus yielding a 14-fold increase in drug sensitivity. When plotted against the C_6_-SM concentration, it is evident that essentially no further decreases in doxorubicin EC_50_ occurred above 6 *μ*M of lipid (insets of Figures 4a and b), and that low micromolar concentrations were relatively most beneficial in this respect. Similar results were obtained with MCF-7 and KB cells (not shown). C_6_-SM itself was virtually nontoxic at the presently used concentrations and incubation periods (not shown).

To examine which molecular features of C_6_-SM are critical for its doxorubicin uptake-enhancing properties, we tested a series of closely related (synthetic) lipid analogues (Table 1

**Table 1:** Doxorubicin uptake-enhancing effect is an exclusive property of truncated, phosphocholine-containing sphingolipids

**Lipid (10 *μ*M)**	**Doxorubicin (% of control±s.d.)**
Control (no lipid, no ethanol)	100.0±17.0
C_6_-sphingomyelin	328.3±24.4
C_8_-ceramide 1-phosphate	116.5±20.6
C_6_-ceramide	105.6±6.5
Sphingosinephosphorylcholine	143.2±10.0
C_2_-sphingomyelin	129.6±19.6
C_6_-phosphatidylcholine	101.0±28.2
Vehicle control (1% v v^−1^ ethanol)	91.5±4.8

A measure of 10 *μ*M of the indicated lipid analogue was administered to BAEC in combination with 50 *μ*M of doxorubicin. After 60 min, cellular drug content was determined fluorimetrically after washing and lysis in 1% (v v^−1^) TX-100. All values were corrected for protein contents and data are expressed as mean percentage to control (no lipid, no ethanol) (±s.d., *n*=3). C_6_-SM had little effect on the fluorescent properties of doxorubicin and hence its quantification (50 *μ*M of doxorubicin yielded fluorescence levels of 93906±793 in the presence of 10 *μ*M C_6_-SM *vs* 86019±1060 in the absence of the lipid). Furthermore, C_6_-SM did not induce any interaction between doxorubicin and plastic cell culture ware (only 6.2±4.4% of additional residual plastic-associated doxorubicin in the presence of 10 *μ*M C_6_-SM).

), in an assay similar as employed for C_6_-SM. We established that short-chain sphingolipid analogues without a choline or phosphocholine head group moiety (C_8_-ceramide-1-phosphate and C_6_-ceramide, respectively) had little or no effect. Sphingosylphosphorylcholine (C_0_-SM) and C_2_-SM exhibited a moderate effect, whereas short-chain phosphatidylcholine, a glycerol-containing lipid, was completely without effect. Thus, the observed effect on drug uptake is optimal for phosphocholine-containing truncated sphingolipid analogues, but the *N-*acyl truncation should not be too rigorous and probably not too small either, since supplementation of natural long-chain SM does not work.

### C_6_-SM does not act by inducing membrane leakage, endocytosis or efflux inhibition

Plasma membrane integrity was monitored by measuring the uptake of the fluid phase marker hydrazide-AF488 by BAEC. No additional inward flux of this membrane-impermeant marker was observed in the presence of 10 *μ*M C_6_-SM (97.4±18.2% uptake, as compared to 100% control uptake). Conversely, no C_6_-SM-induced release of the cytosolic enzyme LDH was observed (19.0±3.9% of total LDH activity released into the culture medium within an hour under control conditions, and 19.7±3.5% in the presence of 10 *μ*M C_6_-SM). These data are consistent with each other, indicating that C_6_-SM leaves the plasma membrane physically intact.

We investigated whether C_6_-SM stimulates the endocytic machinery in our system by using fluorescent conjugates of transferrin and the CTB subunit, as markers for clathrin- and raft-mediated endocytosis, respectively (Mellman, 1996; Orlandi and Fishman 1998). No increased uptake of fluorescence by BAEC in the presence of the lipid was observed. Instead, a small decrease was detectable (67±36% of transferrin and 80±23% of CTB uptake, as compared to 100% uptake under control conditions). Moreover, when cells were maintained at 4°C or were depleted for ATP (with sodium azide and deoxyglucose; Schwoebel *et al*, 2002), conditions that block endocytosis, the C_6_-SM-stimulated doxorubicin uptake remained unaffected. Thus, we conclude that the C_6_-SM effect is unrelated to endocytosis.

Although the presently used cells express only modest levels of drug transporter proteins, such as MDR1 and MRP1, we tested for the possible involvement of these proteins in the observed C_6_-SM effect on doxorubicin uptake. Three different experimental approaches were employed (Table 2

**Table 2:** ABC-transporter proteins are not involved in C_6_-SM-enhanced doxorubicin uptake

**Cell type**	**C_6_-SM 10 *μ*M**	**ATP depletion**	**PSC833 5 *μ*M**	**MK571 12.5 *μ*M**	**Mrp1^−/−^ Mdr1a^−/−^ Mdr1b^/−^**	**Doxorubicin (% of control±s.d.)**
BAEC	−	−	−	−	−	100.0±28.5
	+	−	−	−	−	369.0±113.0
	−	+	−	−	−	108.2±46.7
	+	+	−	−	−	410.9±157.0
	−	−	+	−	−	76.0±15.6
	+	−	+	−	−	278.1±40.7
	−	−	−	+	−	103.5±30.2
	+	−	−	+	−	323.3±23.0
						
Kidney fibroblasts	−	−	−	−	−	100.0±4.4
	+	−	−	−	−	331.8±50.2
	−	−	−	−	+	101.7±14.3
	+	−	−	−	+	385.1±24.7

Doxorubicin accumulation was monitored in the indicated cell types during 60 min, in the absence or presence of 10 *μ*M C_6_-SM. Cells were ATP-depleted during a 90 min preincubation in glucose-free culture medium, supplemented with 10 mM 2-deoxy-D-glucose and 10 mM sodium azide. Alternatively, 5 *μ*M PSC833 or 12.5 *μ*M MK571 was administered to the cells at least 15 min before exposure to doxorubicin. Kidney fibroblasts were isolated from control mice or from Mrp1/Mdr1a/Mdr1b triple knockout mice. All data are expressed as mean percentages±s.d. (*n*=3) to the untreated controls of either BAEC or fibroblasts.

). (1) Since ABC-transporters are energy-dependent, we used a regimen of sodium azide and deoxyglucose that eventually depletes cells from ATP (Schwoebel *et al*, 2002). Under these conditions, and in the absence of C_6_-SM, no accumulation of doxorubicin in BAEC was detected above control levels. In addition, no significant increase in the C_6_-SM effect was observed. (2) PSC833 and MK571 are inhibitors of MDR1 and MRP1, respectively (Mayer *et al*, 1997; Kok *et al*, 2000). At their effective concentrations, no additional accumulation of doxorubicin was observed in the absence of C_6_-SM, and no further increase was observed in the presence of C_6_-SM, not even in combination with ATP depletion (not shown). (3) Like in normal fibroblasts, the doxorubicin uptake-enhancing effect of C_6_-SM was observed in fibroblasts that we derived from *Mrp1/Mdr1a/Mdr1b* triple knockout mice (a three- to four-fold increase, as compared to doxorubicin uptake in the absence of the lipid analogue) (Wijnholds *et al*, 2000). Collectively, the data obtained from these metabolic, pharmacological and genetic approaches (Table 2) allow us to conclude that the C_6_-SM effect on doxorubicin accumulation is unrelated to ABC-transporter proteins.

### C_6_-SM inserts into the plasma membrane but its effect is independent of lipid rafts

Within 60 min, only an estimated 1.5% of the total added amount of C_6_-SM (at 10 *μ*M) was taken up by BAEC, reflecting the relative water-solubility of the truncated lipid. When compared to the cellular pool of endogenous SM (13.15±3.96 nmol mg^−1^ of protein), this fraction of inserted C_6_-SM was nevertheless substantial (1.81±0.47 nmol mg protein^−1^). This cell-associated C_6_-SM was fully responsible for the enhanced doxorubicin uptake, since the effect remained after washing away C_6_-SM that had not been taken up by the cells. The drug uptake-enhancing effect was strongly diminished when membrane-inserted C_6_-SM was hydrolysed by exogenously applied bSMase, or by extraction by a BSA back-exchange procedure (not shown). Since bSMase and BSA act from outside at the cell surface and have no access to the cell interior, these results indicate that C_6_-SM exerts its effect in the outer leaflet of the plasma membrane lipid bilayer.

Given the fact that natural long-chain SM is highly enriched in lipid rafts, we questioned whether C_6_-SM would likewise accumulate into these lipid microdomains. To answer this question, we employed an established isolation procedure, based on the insolubility of these membrane microdomains in ice-cold Triton X-100, combined with ultracentrifugation on a discontinuous sucrose gradient (Lisanti *et al*, 1995). As expected, natural SM was highly enriched in this DRM fraction (Table 3

**Table 3:** No preferential insertion of C_6_-SM into lipid rafts

**Lipid**	**Total**	**DRM**	**non-DRM**
Endogenous SM	13.15±3.96	11.86±1.26	2.87±0.34
Inserted C_6_-SM	1.81±0.47	0.22±0.17	1.16±0.56

BAEC were incubated for 60 min with 10 *μ*M C_6_-SM. *DRM* were then isolated by solubilisation of the cells in ice-cold Triton X-100, and a subsequent ultracentrifuge run on a discontinuous sucrose gradient (see *Materials and Methods*). The initial Triton X-100 suspension was designated as *total*. The upper half of the gradient was designated as *DRM*, and the lower half as *non-DRM*. Total lipids were extracted from all three samples and glycerol-containing lipids were removed by a mild alkaline hydrolysis procedure. The remaining lipids were then separated by TLC and visualised by iodine vapour staining. C_6_-SM and endogenous SM containing spots were scraped from the plate and subjected to a colorimetric phosphate determination. Lipid contents are expressed as nmol mg^−1^ of total cellular protein (±s.d., *n*=3).

). C_6_-SM, on the other hand, was predominantly present in the *non-DRM*. The distribution of endogenous SM in control cells was identical to that of C_6_-SM-treated cells, indicating that no jostling of natural SM occurred by its truncated counterpart. To exclude the possibility that low temperatures and the use of a detergent like Triton X-100 induced artefactual changes in the distribution of (short-chain) lipids, we repeated the experiment with Brij 98, allowing raft isolation at room temperature (Pike *et al*, 2002). However, the distribution of both natural and short-chain SM over the *DRM vs non-DRM* fractions was identical to those of the Triton X-100 isolation (not shown).

We investigated whether the doxorubicin uptake-enhancing effect of the lipid might nevertheless be functionally dependent on lipid rafts. We therefore, in BAEC, strongly reduced the levels of cholesterol, SM and glycosphingolipids, the major lipid constituents of rafts, by employing CDX, bSMase and PPMP, respectively (Radin *et al*, 1993; Ohvo and Slotte, 1996; Veldman *et al*, 1998). Although each of these compounds exhibits a strong impact on the integrity and function of lipid rafts, none of these induced effects on doxorubicin uptake itself, nor on the C_6_-SM-enhancing effect on this (Table 4

**Table 4:** C_6_-SM effect on doxorubicin uptake by BAEC is independent of natural sphingomyelin and other lipid raft components

**C_6_-SM 10 *μ*M**	**CDX 10 mM, 30 min**	**bSMase 200 mU ml^−1^, 2 h**	**PPMP 30 *μ*M, 96 h**	**Doxorubicin (% of control±s.d.)**
−	−	−	−	100.0±13.4
+	−	−	−	353.3±62.6
−	+	−	−	113.0±28.1
+	+	−	−	264.7±72.1
−	−	+	−	104.3±14.2
+	−	+	−	386.2±74.7
−	−	−	+	166.5±22.5
+	−	−	+	340.7±78.6

Cells were pretreated by 10 mM CDX for 30 min, 200 mU ml^−1^ bSMase for 2 h or 30 *μ*M PPMP for 96 h. The doxorubicin uptake-enhancing effect of C_6_-SM was then established. Data are expressed as percentage (±s.d., *n*=3) to control (no pretreatment, no lipid).

).

### C_6_-SM acts optimally on amphiphilic compounds

**Figure 5:**
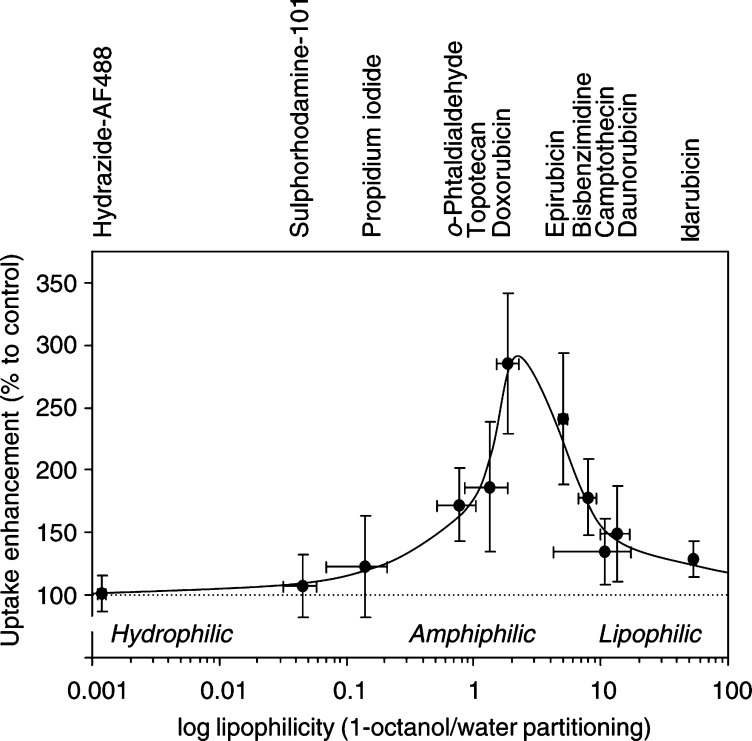
C_6_-SM uptake enhancement is optimal for amphiphilic compounds. During a 60 min period, confluent BAEC were allowed to take up one of a series of fluorescent compounds, either in the absence (control) or presence of 10 *μ*M C_6_-SM. After washing, drug accumulation was determined by fluorimetric analysis of the cell lysates. The C_6_-SM effect on cellular accumulation of the compounds is expressed as percentage to the controls (mean±s.d., *n*=4). These data are plotted against the (log) lipophilicity of each compound, as determined by its partition ratio over a two-phase system consisting of 1-octanol and water (mean±s.d., *n*=4).

In addition to doxorubicin, we tested an array of fluorescent chemotherapeutic drugs and intracellular probes for their sensitivity towards C_6_-SM-uptake enhancement. In addition, we determined the lipophilicity of each of these compounds by establishing their partitioning ratio (*P*) over a defined 1-octanol/water system (Washington *et al*, 2001). When these parameters were plotted against each other (Figure 5

), a clear optimum was observed in the *P* range of 1–6 (amphiphilic to slightly lipophilic). In addition to doxorubicin, other amphiphilic drugs such as epirubicin and topotecan were identified as being C_6_-SM sensitive as well. In contrast, both water-soluble compounds and very lipophilic drugs such as camptothecin, daunorubicin and idarubicin were mostly insensitive to C_6_-SM.

## DISCUSSION

We discovered here that the short-chain sphingolipid analogue C_6_-SM facilitates the cellular influx of amphiphilic drugs *in vitro*, in turn leading to an enhanced cytotoxicity. This effect was most pronounced for doxorubicin. This anthracycline showed optimal lipophilicity, which turned out to be a decisive factor in the C_6_-SM-facilitated uptake. The lipophilicity of chemically related anthracyclines daunorubicin and idarubicin is significantly higher, which correlated with less effect. The drug uptake-enhancing effect turned out to be highly specific for C_6_-SM, since even minor modifications in this sphingolipid structure largely diminished the effect on drug uptake. At its active concentrations (2–6 *μ*M), C_6_-SM itself was virtually nontoxic to cells. Therefore, this lipid can be considered as a novel potentiator of doxorubicin cytotoxicity.

The precise mechanism by which C_6_-SM enhances drug uptake remains unestablished, but we ruled out the following five possibilities: (1) C_6_-SM did not induce nonspecific membrane damage, for example by exhibiting detergent-like effects. By (local) dissolving of the plasma membrane, this would have resulted in an increased drug influx. However, at the concentrations used in this study, C_6_-SM did not increase the membrane permeability of a hydrophilic marker molecule (AF488-hydrazide) or a cytosolic protein (LDH), indicating that plasma membrane integrity was indeed maintained. (2) The enhanced doxorubicin uptake by C_6_-SM was not due to an increase in endocytic activity. Although doxorubicin is known to traverse the plasma membrane barrier by passive diffusion along its concentration gradient, it is conceivable that some internalisation may also occur along with (constitutive) endocytosis, and that C_6_-SM might stimulate this route. In *S. cerevisiae*, for example, certain sphingolipids are involved in endocytotic signalling processes (Friant *et al*, 2001). However, C_6_-SM did not stimulate the cellular uptake of transferrin and CTB, markers for, respectively, clathrin-dependent and clathrin-independent endocytic routes. In addition, C_6_-SM-enhanced doxorubicin uptake also occurred at low temperatures and was ATP-independent. Together, these results exclude any involvement of endocytic processes. (3) The effect of C_6_-SM did not result from an interaction with members of the ATP-binding cassette super family of transporter proteins (ABC transporter proteins), which are able to mediate the efflux of anthracyclines like doxorubicin against a concentration gradient (Borst and Oude Elferink 2002). Some ABC transporter proteins, such as MDR1 and MRP1, have the capacity to translocate lipids (Smit *et al*, 1993; van Helvoort *et al*, 1996; Schmitz *et al*, 2000). Since intracellular drug concentrations are determined by a balance between drug influx and efflux, C_6_-SM might have indirectly caused a doxorubicin accumulation by inhibition of the cellular efflux capacity. However, neither metabolic, nor pharmacological, nor genetic intervention with the function of ABC transporter proteins affected the C_6_-SM-enhanced doxorubicin uptake. (4) No modification of doxorubicin by C_6_-SM occurs before membrane insertion. The uptake-enhancing effect was retained when the lipid and drug were presented to cells after each other, with extensive washing in between. This observation indicates that no complex formation between the two occurred prior to plasma membrane association. Therefore, no possible lipid-induced changes in the physicochemical properties of doxorubicin can explain its increased uptake efficiency. (5) Natural lipid rafts play no role in the C_6_-SM-enhanced drug uptake. While natural long-chain SM is highly enriched in lipid rafts (Veldman *et al*, 2001), its short-chain analogue, C_6_-SM, did not accumulate in these membrane microdomains (at least not in the *DRM* fraction). Furthermore, artificial disruption of these microdomains, by either cholesterol extraction, SM hydrolysis or glycosphingolipid depletion, had no effect on the doxorubicin uptake-enhancing effect of C_6_-SM.

Having excluded the above possibilities, what other clues do we have to explain the lipid-enhanced drug uptake? Firstly, the C_6_-SM effect is abrogated when the lipid is removed by BSA back-extraction (simple washing with buffer is ineffective) or hydrolysed by treatment with an exogenous bacterial SMase. Since BSA and bSMase have no direct access to the cell interior, these results indicate that C_6_-SM acts at the outer leaflet of the plasma membrane. Furthermore, only a small fraction (1.5%) of the supplied C_6_-SM is taken up by cells. This finding is consistent both with its relative hydrophilicity and with the observed plateau in doxorubicin-uptake enhancement and cytotoxicity, reached at about 8 *μ*M of C_6_-SM (Figures 3 and 4). This possibly indicates a saturation of particular membrane insertion sites. Once in the membrane, C_6_-SM might not be randomly dispersed but, like natural long-chain sphingolipids, show a tendency to self-aggregate in the plane of the membrane (van Blitterswijk *et al*, 1987, 2003; Holopainen *et al*, 1998). It is therefore appealing to speculate that these physical properties allow C_6_-SM to form their own ‘artificial’ rafts, which, however, remain detergent soluble (as found here). In this way, and by virtue of its acyl truncation, it may be speculated that C_6_-SM induces local ‘voids’ in the lipid bilayer that could accommodate doxorubicin molecules. Owing to the resulting local changes in lipophilicity, thickness and fluidity, the plasma membrane permeability for amphiphilic drugs would then specifically be facilitated. Alternatively, self-aggregation of C_6_-SM might lead to artificial ‘channels’ in the membrane, providing a preferential gateway for drug uptake, similar as described, for example for ceramides (Siskind and Colombini, 2000; Siskind *et al*, 2002). Unfortunately, the possible formation of distinct C_6_-SM rafts or channels is difficult to prove experimentally at present. Natural lipid rafts can be isolated as a *DRM* fraction, but C_6_-SM is excluded from this fraction. Further investigation of this issue therefore awaits the development of more sophisticated (detergent-free) isolation procedures.

Since the present *in vitro* findings seem promising, it is of interest to speculate on possible *in vivo* applications. Of all drugs tested so far, doxorubicin turned out to be the most sensitive towards the uptake-enhancing properties of C_6_-SM. We used doxorubicin at a concentration of 50 *μ*M, which corresponds well to common plasma concentrations in patients upon a single bolus administration of 40–60 mg m^−2^ body surface. The doxorubicin uptake-enhancing effect of C_6_-SM was particularly strong within 15–60 min of drug administration. Since the *t*_1/2_ clearance of systemically applied doxorubicin is approximately 1.25 h, this is a very relevant interval, which would allow target tissues to accumulate larger amounts of the drug (Robert and Gianni, 1993). Theoretically, significant lower doses of doxorubicin would thus be required to obtain a successful (clinical) outcome, when coadministered with the short-chain sphingolipid. However, to actually improve the therapeutic efficacy of doxorubicin by coadministration of C_6_-SM, some advantageous (tumour) tissue selectivity of the lipid is required. In this context, promising preliminary results were obtained with cultured rat cardiac myoblasts. For as yet unknown reasons, these cells are not susceptible towards the sphingolipid analogues (Veldman *et al*, unpublished observation). Given the fact that cardiotoxicity is an important limiting factor in anthracycline-based chemotherapy, this result is encouraging.

On the other hand, some open questions remain with respect to the usefulness of systemically applied short-chain SM. Namely, due to its affinity for serum components, such as albumin and lipoproteins, or due to association with nonrelevant cell membranes (e.g. erythrocytes), large amounts of the lipid might be needed to reach the required plasma concentrations. Potential hazards then include unwanted toxicity for vessel walls or other nontumour tissues/cells. Furthermore, the possibility exists that C_6_-SM and doxorubicin (or any other amphiphilic drug) differ in pharmacokinetics, biodistribution and metabolism, and may thus not be delivered at the same time at the same site, which is a prerequisite for the drug uptake-enhancing effect. Extensive pharmacological studies into the toxicity, efficacy, pharmacokinetics and bioavailability of C_6_-SM (in combination with amphiphilic drugs or not) are therefore required to assess the feasibility of clinical C_6_-SM-based drug delivery. Hopefully, our present *in vitro* data will encourage new experiments in this direction.
